# A Versatile Drift‐Free Super‐Resolution Imaging Method via Oblique Bright‐Field Correlation

**DOI:** 10.1002/advs.202412127

**Published:** 2024-12-24

**Authors:** Hongqiang Ma, Phuong Nguyen, Yang Liu

**Affiliations:** ^1^ Department of Bioengineering Beckman Institute for Advanced Science and Technology University of Illinois Urbana‐Champaign Urbana IL 61801 USA; ^2^ Department of Bioengineering Department of Electrical and Computer Engineering Beckman Institute for Advanced Science and Technology Cancer Center at Illinois University of Illinois Urbana‐Champaign Urbana IL 61801 USA

**Keywords:** drift correction, super‐resolution microscopy

## Abstract

High‐resolution optical microscopy, particularly super‐resolution localization microscopy, requires precise real‐time drift correction to maintain constant focus at nanoscale precision during the prolonged data acquisition. Existing methods, such as fiducial marker tracking, reflection monitoring, and bright‐field image correlation, each provide certain advantages but are limited in their broad applicability. In this work, a versatile and robust drift correction technique is presented for single‐molecule localization‐based super‐resolution microscopy. It is based on the displacement analysis of bright‐field image features of the specimen with oblique illumination. By leveraging the monotonic relationship between the displacement of image features and axial positions, this method can precisely measure the drift of the imaging system in real‐time with sub‐nanometer precision in all three dimensions, over a broad axial range, and for various samples, including those with closely matched refractive indices. The performance of this method is validated against conventional marker‐assisted techniques and demonstrates its high precision in super‐resolution imaging across various biological samples. This method paves the way for fully automated drift‐free super‐resolution imaging systems.

## Graphical abstract

This study introduces a robust marker‐free drift correction method for single‐molecule localization‐based super‐resolution microscopy. Utilizing oblique illumination, it analyzes bright‐field image feature displacement to achieve sub‐nanometer precision in real‐time drift tracking across all dimensions. Validated against conventional techniques, this versatile approach supports automated drift‐free imaging, ensuring nanoscale precision for prolonged high‐resolution microscopy in diverse biological samples.

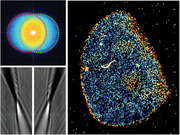

## 1 Introduction

Single‐molecule localization microscopy (SMLM), such as Photoactivated Localization Microscopy (PALM),^[^ ^1^, ^2^ ^]^ Stochastic Optical Reconstruction Microscopy (STORM),^[^ ^3^, ^4^ ^]^ and DNA points accumulation for imaging in nanoscale topography (DNA‐PAINT),^[^ ^5^, ^6^ ^]^ has revolutionized optical imaging by breaking the diffraction limit and enabling the observation of biological structures at nanometer‐scale resolution. Its simple wide‐field configuration, coupled with superior resolution, makes SMLM appealing for many applications. However, SMLM typically requires the accumulation of single molecules across tens of thousands of frames to reconstruct the final image. However, the prolonged image acquisition time makes SMLM susceptible to sample drift caused by thermal fluctuation, mechanical vibration, and other environmental factors, which can significantly degrade the resolution of the reconstructed images.^[^ ^7^ ^]^ To address this problem, numerous post‐processing drift correction algorithms have been developed,^[^ ^8^, ^9^, ^10^, ^11^ ^]^ though their effectiveness is limited to small axial drift within the depth of field of SMLM. As a result, an online hardware‐based active drift correction method is essential for maintaining sample focus and ensuring robust and high‐precision super‐resolution imaging.^[^ ^7^ ^]^ Integrating a reliable hardware drift correction module in the SMLM system becomes one of the most challenging aspects of the system.

Existing online drift correction methods can be broadly categorized into two main types. The first is the fiducial marker‐assisted drift tracking method,^[^ ^12^, ^13^, ^14^, ^15^ ^]^ which is widely used in the SMLM system and considered as the gold standard for its nanoscale precision. However, this approach presents a significant drawback: it complicates sample preparation by requiring that fiducial markers do not interfere with the sample and are distributed at the proper density within the imaging field of view (FOV). Moreover, the non‐uniform distribution of markers and the complexity of data analysis make fully automated imaging workflow difficult to achieve.

The second type comprises marker‐free drift tracking techniques, including monitoring the reflection of the illumination beam^[^ ^16^, ^17^, ^18^, ^19^, ^20^ ^]^ and bright‐field image cross‐correlation.^[^ ^21^, ^22^, ^23^, ^24^ ^]^ Among these, reflection beam monitoring is particularly popular in high‐resolution microscopy systems for automated focus maintenance, as seen in Nikon's Perfect Focus system.^[^ ^16^ ^]^ Unlike marker‐assisted methods, these approaches do not require special sample preparation. Instead, it tracks imaging focus by detecting the position shift or shape changing of reflected light at the interface between the specimen and the coverslip. While effective in cases with a significant refractive index mismatch, their performance declines when such refractive index difference between the sample and the coverslip is small—a common scenario in high‐resolution microscopy to minimize spherical aberration or when biological cells are closely attached to the coverslip surface. Furthermore, this method is unable to track the drift in the lateral dimension, which is important for‐high‐precision super‐resolution imaging.

On the other hand, the bright‐field image correlation approach utilizes the label‐free structural features of the specimen, obtained from bright‐field microscopy images, to track drift without interfering with the sample itself or requiring system modifications.^[^ ^25^ ^]^ However, this method is not widely adopted for automatic focusing on high‐resolution microscopy systems, as bright‐field images are often susceptible to factors such as uneven illumination, low contrast of specimen features, and out‐of‐focus artifacts. To achieve acceptable precision (6–20 nm), users must manually select a small, high‐contrast region of interest (ROI) for each experiment, significantly limiting the broad applicability of the method.^[^ ^21^, ^22^ ^]^ Furthermore, this approach typically maintains a reliable monotonic relationship between correlation coefficients and defocus positions only within a narrow axial range (−300–300 nm),^[^ ^21^, ^22^ ^]^ making it unreliable for correcting large drifts.

Here, we present a versatile, marker‐free drift correction method that addresses the limitations of existing drift correction techniques, offering significantly improved precision and robustness for a broader range of biological samples and imaging conditions. Our method leverages the label‐free nature of bright‐field image features but, unlike previous approaches, it utilizes oblique illumination at two complementary angles. This configuration enables precise lateral displacement analysis to accurately determine the axial position. To further improve the reliability of bright‐field images, we apply feature enhancement processing, which improves the contrast of specimen features, mitigates the effects of nonuniform illumination and out‐of‐focus artifacts, and achieves sub‐nanometer precision in all three dimensions. Additionally, our method exhibits superior robustness by maintaining a high‐precision, monotonic relationship over an extended axial range of more than 20 µm. We demonstrate that our method can be efficiently integrated into SMLM with minimal system modification, showing its efficacy in super‐resolution imaging across various biological samples, ranging from cells to tissue slices, even in cases of closely matched refractive indices.

## 2 Results

### 2.1 Principle of Drift Correction via Oblique Bright‐Field Correlation

Tracking the label‐free sample features using bright‐field imaging represents a simple and convenient method for 3D online drift correction. However, transparent samples used in SMLM typically exhibit low structural contrast in the bright‐field images, making them susceptible to various background signals, such as nonuniform illumination, coherent diffraction patterns from contaminants and debris in the imaging medium, or on the optics and coverslip.

In this study, we introduced a bright‐field displacement‐based drift correction method for the SMLM imaging system. In principle, in bright‐field imaging, a sample illuminated by a plane wave produces a total field that is the interferometric superposition of the incident plane wave and the scattered wave from the sample. According to the Fourier diffraction theorem, the scattered field encodes the spatial frequency information of the object. As shown in Figure S1 (Supporting Information), the oblique illumination angle shifts the spatial frequency spectrum in the Fourier domain, thus introducing the angle‐dependent spatial frequency components and tilting the point spread function of the imaging system.

**Figure 1:**
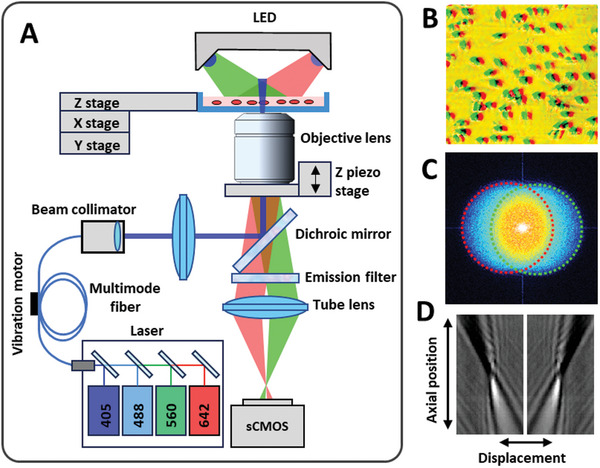
The schematic of our drift‐free imaging method via displacement analysis of bright‐field image features from oblique illumination. A) Schematic of our optical imaging system. The displacement of the bright‐field image features from the same sample under a pair of oblique illuminations (indicated as Red and Green) varies with the axial position. B) A merged bright‐field image illustrating the displacement between features under the two oblique illuminations (indicated as Red and Green). C) The phase shift of the paired images in the Fourier domain. D) The monotonic relationship between the lateral displacement of the oblique point spread function and the axial position.

To robustly implement this method in the SMLM system, we used two new strategies. First, we used oblique bright‐field illumination to establish a linear relationship between the axial position of the sample and the translational displacement of its structural features (**Figure** **1**). The bright‐field images from a pair of oblique, partially coherent illuminations with complementary angles (Figure 1A) exhibit a lateral shift when the sample is out of focus (Figure 1B).^[^ ^26^ ^]^ This approach is based on the general principle that the defocused images from the paired oblique illumination exhibit a phase shift in the Fourier domain (Figure 1C), which corresponds to a lateral shift in the image plane,^[^ ^25^ ^]^ as described in detail in the Experimental Section. As a result, the axial drift can be accurately determined by measuring the translational displacement of the two oblique bright‐field images (Figure 1D). The displacement is calculated based on identifying the position corresponding to the peak value in the cross‐correlation map of the two bright‐field images. This approach encodes the axial position with spatial displacement rather than the correlation coefficient itself, enabling further image processing to enhance the contrast of the image features.

In our current implementation, we adopted a sequential image acquisition and drift correction strategy where the drift correction was performed intermittently at intervals of 500 frames (15 s) during image acquisition. This approach was chosen to minimize modifications to the existing imaging system. Alternatively, as shown in Figure S2 (Supporting Information), a parallel strategy can be implemented by integrating an independent drift correction module equipped with a dedicated camera. This strategy would enable real‐time drift correction without interfering with fluorescence image acquisition.

**Figure 2:**
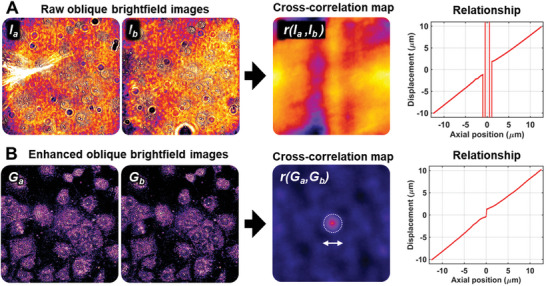
Comparison of the contrast of the oblique bright‐field images and their cross‐correlation map before A) and after B) feature enhancement processing. The enhancement process significantly improves the robustness in finding the peak value in the cross‐correlation map, which directly determines the relationship between feature displacement and axial position.

Second, to enhance the robustness and precision in determining the lateral displacement between two oblique bright‐field images, we implemented a feature enhancement pre‐processing step. As shown in **Figure** **2**A, the high‐frequency shot noise and low‐frequency background noise due to uneven illumination and out‐of‐focus image artifacts can significantly reduce the accuracy of determining the lateral displacement between two oblique bright‐field images, especially when the sample is close to the focal plane. To address this issue, as shown in Figure 2B, we applied a bandpass filter to enhance the contrast of the image by suppressing both the high‐frequency shot noise and low‐frequency background noise. This approach selectively improves the contrast of bright‐field features from biological structures, thus allowing the use of the full bright‐field image over the entire field of view (FOV), rather than relying on a manually cropped small ROI to estimate drift. These two aspects significantly improve the reliability and precision of bright‐field correlation‐based drift correction to the nanometer scale.

### 2.2 Performance Evaluation of Our Method

**Figure 3:**
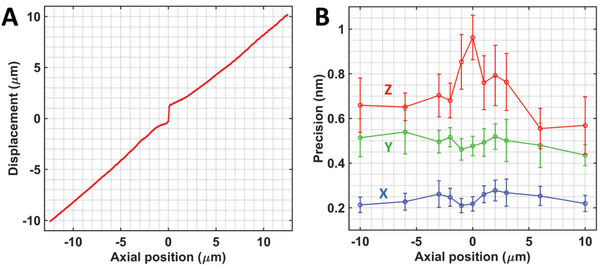
The monotonic relationship A) between the lateral feature displacement and axial position and the 3D drift estimation precision B) across a large axial range of 25 µm.

We first validated the monotonic relationship between the lateral displacement of image features and the axial position of the sample on imaging the commonly used COS‐7 cells. We measured the lateral displacement of the two bright‐field images under oblique illumination (±45°) over a long range of axial positions between −12.5 and 12.5 µm with a step size of 100 nm. As shown in **Figure** **3**A, there is a monotonic relationship with a slope ratio of ≈0.78 for the illumination angle (±45°). We noticed a discontinuity near the focal plane along the near‐linear relationship, which could be caused by the asymmetric nature of the bright‐field point spread function (Figure S3, Supporting Information).

Next, we evaluated the precision of drift estimation of our method in all three dimensions by performing multiple measurements over a long range of axial positions of 20 µm. We took 20 continuous measurements at each position, and the standard deviation of these 20 independent measurements was used as the precision. At each position, we repeated five experiments, and the mean and variation values of the precision from these five repeated experiments are illustrated in Figure 3B. The measured precision is smaller than 0.3, 0.6, and 1.0 nm in the x, y, and z dimensions, respectively. Compared to traditional bright‐field correlation methods,^[^ ^21^, ^22^ ^]^ our approach achieves a significantly improved performance over an order of magnitude in both precision and axial range. The results also indicate that the precision improves when the sample is positioned at a larger defocused distance. Therefore, utilizing a larger defocused position (e.g., 5 µm) for drift measurement as the reference point can enhance the accuracy of drift correction.

### 2.3 Performance Benchmarking Against Marker‐Assisted Drift‐Correction Method

**Figure 4:**
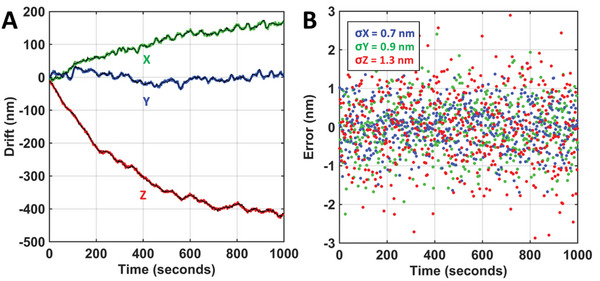
Comparison of the drift correction performance of our method and fiducial marker‐assisted method. A) Comparison of the estimated 3D drift trajectories. B) Overall estimation error of our method including piezo movement error.

We then evaluated the performance of our drift correction method by comparing it against the fiducial marker‐based methods, also on COS‐7 cells. The fiducial markers (nanodiamond, 100 nm) were randomly placed on the surface of the cover glass. We selected ≈20 well‐isolated markers with an average intensity of 20 000 photons, which is expected to achieve a sub‐nanometer localization precision. We performed drift estimation and correction using our approach every two seconds and then used the fiducial markers to estimate the drift correction error. As shown in **Figure** **4**A, the drift trajectories estimated by the two methods are almost identical, exhibiting a standard deviation of less than 1 nm in the lateral dimension and ≈1.3 nm in the axial dimension (Figure 4B). This experiment demonstrates that in samples with a strong refractive index mismatch between the sample and the coverslip, our method can track 3D drift at a comparable precision to the gold standard of the fiducial marker‐based method over a long axial range.

### 2.4 Experimental 2D STORM Imaging of Cells and Tissue Section

**Figure 5:**
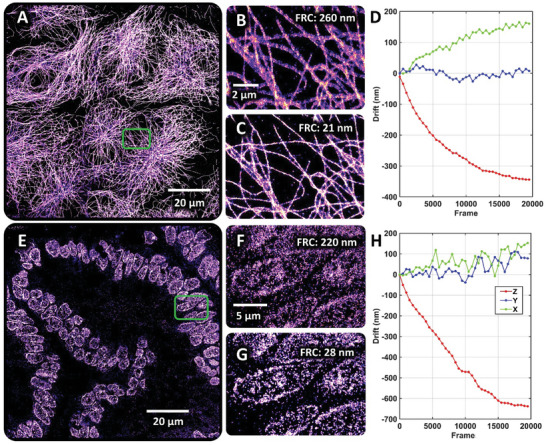
Super‐resolution imaging performance with our drift‐free imaging method. A) STORM image of microtubules in COS‐7 cells. B,C) The zoomed region of (A) without and with lateral drift correction, and D) the 3D drift trajectories retrieved by our method. E) STORM image of heterochromatin (immunofluorescently labeled against H3K9me3) in a colon tissue section. STORM image of microtubules on COS‐7 cells. F,G) The zoomed region of (E) without and with lateral drift correction and H) the 3D drift trajectories estimated by our method.

We further evaluated the performance of our drift correction method for super‐resolution imaging of microtubules on COS‐7 cells (**Figure** **5**A–D). The microtubules in fixed COS‐7 cells were labeled with Alexa Fluor 647 and imaged using 2D SMLM (Figure 5A). We performed online drift correction on the axial dimension (≈350 nm axial drift) during the data acquisition of SMLM imaging. The reconstructed super‐resolution image of microtubules achieved a significantly improved FRC resolution,^[^ ^27^ ^]^ from 260 to 21 nm, by compensating for the lateral drift with our drift correction method (Figure 5D).

We also evaluated the performance of our drift correction method for super‐resolution imaging of samples with closely matched refractive index between the sample and the coverslip. As shown in Figure 5E–H, we performed STORM imaging of heterochromatin on formalin‐fixed paraffin‐embedded (FFPE) colon tissue section. This is one of the scenarios where conventional reflection‐based drift correction methods perform poorly due to the closely matched refractive index between the medium and the coverslip. As we previously showed, the use of index‐matching medium (60% 2,2‐thiodiethanol (TDE), n ≈1.47) can significantly reduce the background for SMLM imaging of tissue.^[^ ^28^ ^]^ However, the small refractive index difference between the medium and coverslip significantly reduced the reflection signal by over four orders of magnitude, rendering reflection‐based drift correction methods ineffective. We perform real‐time drift correction over the axial range of ≈650 nm during SMLM imaging and the reconstructed super‐resolution image achieved a significantly improved FRC resolution, from 220 to 28 nm, by compensating the lateral drift with our drift correction method (Figure 5H).

### 2.5 Experimental 3D STORM Imaging

**Figure 6:**
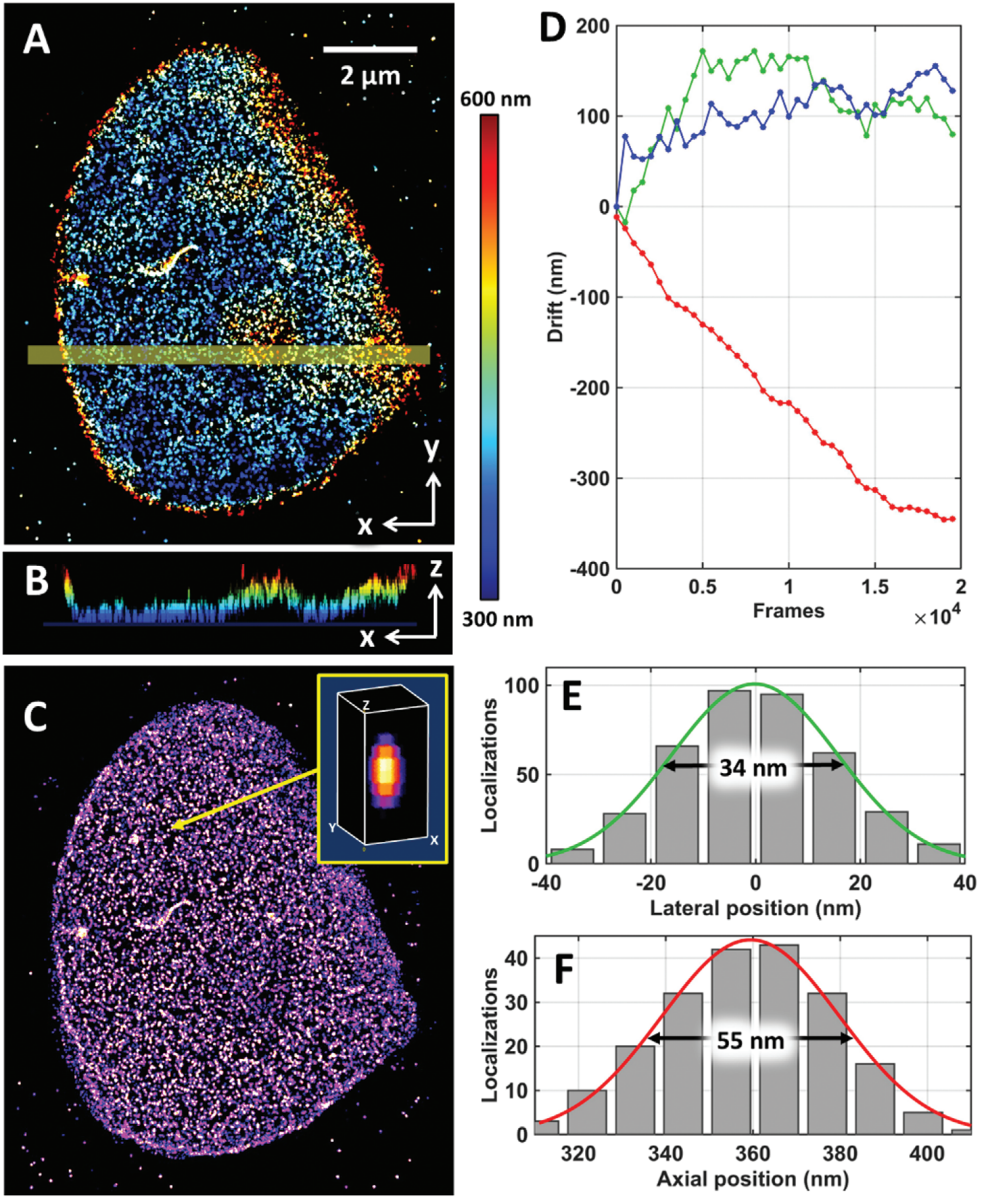
Performance evaluation on 3D super‐resolution imaging of lamin B1. A) The color‐coded 3D STORM image of lamin B1 of COS‐7 cells. B) The x‐z profile of the region in (A). C) The 2D maximum value projection of the 3D STORM image. D) The 3D drift trajectories estimated by our method. E,F) The cross‐sectional profiles of a lamin B1 cluster in the lateral and axial dimensions.

We also evaluated the performance of our drift correction method on 3D super‐resolution imaging of nuclear lamina, using a defocus‐based 3D super‐resolution approach (detailed in Figures S4–S6, Supporting Information). Lamin B1, a nuclear lamina protein, was labeled with Alexa Fluor 647 through immunofluorescence staining in COS‐7 cells. The reconstructed super‐resolution image clearly shows the 3D distribution of lamin B1 clusters and reveals the 3D shape of the nuclear envelope (**Figure** **6**A,B). The size of a lamin B1 cluster exhibits a full‐width half maximum (FWHM) of 34 nm in the lateral dimension and 55 nm in the axial dimension, clearly identifying the dense clusters after compensating for 3D drift with our active drift correction method (Figure 6D). Note that our drift correction method is independent of the specific approach used to implement the 3D SMLM system. Conceptually, the tilt point spread function remains effective for drift correction across various 3D SMLM methods, such as astigmatism, and dual‐plane imaging. Additionally, independent drift correction modules (Figure S2, Supporting Information) can be implemented to enhance the method's applicability without interfering with the fluorescence detection path.

## 3 Discussion and Conclusion

In this study, we developed a robust and versatile active drift correction method for super‐resolution microscopy, based on displacement analysis of bright‐field image features acquired from a pair of oblique illuminations. Our method encodes the axial position of the sample into the translational displacement of these features. By incorporating feature enhancement pre‐processing to mitigate the high‐frequency noise and image artifacts, we significantly improved the precision and the robustness of our displacement‐based drift correction, achieving sub‐nanometer precision, which makes it well‐suited for super‐resolution imaging.

Our method introduces several unique features that enhance its utility in single‐molecule localization microscopy (SMLM), as summarized in **Table** **1**. The first main advantage is its high precision. Unlike conventional active drift correction methods, our approach uses the bright‐field image features of the entire sample in the field of view (FOV) to achieve minimal drift correction errors—sub‐nanometer accuracy in all three dimensions. This enables ultra‐precise super‐resolution imaging of biological structures. The second main advantage is its robustness and broad compatibility. Our method remains effective across a wide axial range, extending up to tens of microns, which is critical for reliable autofocusing.^[^ ^14^, ^21^, ^22^ ^]^ It also maintains consistent focus across various sample conditions, such as refractive index‐matched tissue and high‐density cell samples, where traditional methods often struggle to maintain accurate focus consistently across the entire sample area. Next, our method can also be applied in live‐cell imaging to maintain focus in the axial dimension. Finally, our method ensures practical applicability and ease of adoption. It utilizes the inherent structural features of the sample itself, eliminating the need for special preparation. Adapting this method to the existing supper‐resolution imaging system only requires a pair of LEDs for bright‐field illumination, which is simple to implement and involves less stringent alignment requirements compared to the reflection‐based drift correction methods used for wide‐field SMLM setups. Additionally, the consistent monotonic relationship between axial position and translational displacement of the two oblique bright‐field images across the entire sample allows for fully automated autofocusing, making it possible for whole‐slide imaging and eliminating the need for manual adjustments.

**Table 1:** Performance benchmarking of different active drift correction methods for SMLM.

Utility features		Marker Assisted	Marker‐Free		
			Beam reflection	Bright‐Field Correlation	Our Method
Precision	Lateral dimension	<1 nm	N/A	<10 nm	<1 nm
	Axial dimension	<2 nm	<5 nm	<20 nm	<1 nm
Robustness	Working distance	<1 µm	>20 µm	<1 µm	>20 µm
	Index‐matched sample	Yes	No	Yes	Yes
	Live‐cell imaging	Yes	Yes	No	Yes
Ease to use	Sample preparation	Complex	Easy	Easy	Easy
	Autofocus without user intervention	No	Yes	No	Yes
	System modification	Easy	Complex	Easy	Easy

In conclusion, our active drift correction method greatly improves the robustness of drift‐free super‐resolution microscopy, providing reliable performance, simple implementation, and improved imaging capabilities across a wide range of biological samples, including those with closely matched refractive indices. This online active drift correction approach lays the foundation for fully automated, high‐precision, high‐throughput SMLM imaging systems.

## 4 Experimental Section

### Optical Imaging Setup

The performance of the method was evaluated on this custom‐built high‐throughput SMLM system, which is shown in Figure 2A. The light source consists of four laser lines, including 642 nm (VFL‐P‐1000‐642‐OEM3, 1 W, MPB Communications), 560 nm (2RU‐VFL‐P‐2000‐560‐B1R, 2 W, MPB Communications), 488 nm (DL488‐150, 150 mW, CrystaLaser), 405 nm (DL405‐050, 50 mW, CrystaLaser), respectively. The intensity of each laser was controlled by a neutral density filter (model No. NDC‐50C‐4‐A; Thorlabs, Newton, NJ) counted by a rotational motor. The four laser beams were then combined by the dichroic mirrors and coupled into a multimode fiber with a square core (400 µm, CNI laser). The achromatic collimator (F950SMA‐A, Thorlabs) was used to output the flat‐top square beam. A high‐frequency vibration motor (16 000 RPM) was used to reduce the speckles of the laser beam. The uniform illumination beam was then projected onto the sample by a high‐NA objective lens (UPLXAPO60XO, NA = 1.42, Olympus). For the detection path, the light was collected by the objective lens, passing through a multi‐band dichroic mirror (ZT405/488/561/640rpc, Semrock) and a multi‐band emission filter (ZET405/488/561/640 m, Semrock) and then focused onto the camera by a tube lens (TTL200‐A, Thorlabs). The final image was recorded by an sCMOS camera (ORCA‐Flash4.0 V2, Hamamatsu), corresponding to a pixel size of ≈100 nm on the sample plane. A closed‐loop piezo nanopositioner (Nano‐F100S, Mad City Labs) was used to adjust the axial position of the objective for real‐time drift correction. Data acquisition, laser intensity control, and drift correction were all integrated into the custom‐designed software written in LabVIEW (National Instruments). The field of view was set to be 1024 × 1024 pixels, corresponding to a region of ≈100 × 100 µm^2^.

### Drift‐Correction Module

A pair of blue LEDs (425 nm, 3 W, CHANZON) was employed to illuminate the sample at angles of +45° and −45° to create two oblique bright‐field images. The contrast in the bright‐field images was due to the scattering and refractive index mismatch between different biological structures and their surrounding medium. Because scattering intensity was inversely proportional to the wavelength, shorter wavelength scatters the light more strongly than longer wavelengths. Therefore, the image contrast from the blue light was generally higher in the bright‐field images. Here, blue LEDs were chosen over red LEDs to increase the contrast and resolution of the bright‐field features of the transparent sample. But the proposed drift correction method was not restricted to specific wavelengths. Any wavelength used for bright‐field imaging, including near‐infrared light can also be applied here.

To implement the drift correction approach with minimal modification to the existing SMLM system, a single sCMOS camera was used to capture both oblique bright‐field images for drift monitoring and fluorescence images for SMLM imaging. The LED intensity was controlled by an Arduino board with PWM control. The LED intensity (0.5 mW per field of view) was adjusted to achieve a histogram peak at half of the well capacity of the sCMOS pixel for high SNR bright‐field imaging. This low LED illumination power also helps minimize the risk of fluorescence photobleaching. For SMLM imaging, an exposure time of 20 ms was used with a laser (642 nm) power density of 5 kW cm^−^^2^.

### Image Processing for Drift Correction

A band‐pass filter (ℎ) was first applied to the two bright‐field images (*I_a_* and *I_b_*) under complementary oblique illumination angles to enhance the contrast of biological features and suppress high‐frequency shot noise and low‐frequency background noise. Band‐pass filtering (h) was implemented using a Difference of Gaussians (DoG) operator (σ_1_ = 1 pixel, σ_2_ = 5 pixels for the system) in this implementation. The absolute value was also used to eliminate the contrast reversal effect caused by the asymmetric characteristics of the bright‐field imaging point spread function (PSF).

(1) $$\left\{\begin{array}{c} G_{a}=\left|I_{a}*h\right|\\ G_{b}=\left|I_{b}*h\right| \end{array}\right.$$

Next, the cross‐correlation coefficient map (𝑟_𝑧_) was calculated between the contrast‐enhanced bright‐field images (*G_a_* and *G_b_*) and the cross‐correlation coefficient map (𝑟_xy_) between the sum image (*G_a_* + *G_b_*) and the sum image at the initial time point (*G_a0_* + *G_b0_*) using Fast Fourier Transform‐based equations.

(2) $$\left\{\begin{array}{c} r_{z}=F^{-1}\left\{F\left\{G_{a}\right\}\cdot F^{*}\left\{G_{b}\right\}\right\}\\ r_{xy}=F^{-1}\left\{F\left\{G_{a}+G_{b}\right\}\cdot F^{*}\left\{G_{a0}+G_{b0}\right\}\right\} \end{array}\right.$$

Then, the phase displacement (Δ*z*) and (Δ*x*, Δ*y*) of the paired images can be directly estimated by finding the peak position of the correlation map (𝑟_𝑧_) and (𝑟_xy_).^[^ ^29^ ^]^ Here, the previously developed enhanced phasor‐based localization algorithm^[^ ^17^, ^30^ ^]^ was employed with a window size of 15 × 15 pixels to estimate the peak position with sub‐nanometer precision.

(3) $$\left\{\begin{array}{c} \Delta z=\arg\max_{\left(x,y\right)}\left\{r_{z}\right\}\\ \left(\Delta x,\Delta y\right)=\arg\max_{\left(x,y\right)}\left\{r_{xy}\right\} \end{array}\right.$$

Finally, the lateral drift was estimated as (Δ*x*, Δ*y*) and the axial drift can be determined by referencing the monotonic relationship between the axial position and the translational displacement of the two oblique bright‐field images (Figure 2B). Here an illumination angle of ≈45 degrees was used, and the measured relationship between the axial position and lateral displacement of the two oblique bright‐field images follows a measured ratio of ≈0.78. An example MATLAB code together with representative images was provided in the public repository for reference (https://github.com/YangLiuLab/ObliqueDriftCorr).

### Real‐Time Drift Correction Workflow in the SMlM Imaging System

For each experiment, the focus of the fluorescence target was first identified, and established a simple lookup relationship between phase displacement and three axial positions (−0.1, 0 µm, +0.1 µm). It was then acquired a series of wide‐field fluorescence images (≈20000 frames) at the laser power and imaging buffer for standard SMLM imaging. At appropriate time intervals determined by the stability of the SMLM imaging system, the 3D position of the sample was measured using displacement analysis and compensated for the relative drift in the axial dimension. For most SMLM imaging systems, the drift magnitude was usually less than 500 nm over an experiment over 20 000 frames. The accumulated drift over an interval of 500 frames was typically small enough (< 10 nm) to be further corrected with the post‐processing drift correction algorithms.

### Image Reconstruction and Statistical Analysis

For each dataset, a background correction approach, EVER,^[^ ^31^ ^]^ was first applied to suppress the non‐uniform background. Next, the fast harmonic analysis‐based localization algorithm^[^ ^17^ ^]^ was used to precisely estimate the position of each candidate molecule. Then it was excluded overlapping molecules within a distance of fewer than 5 pixels and dim molecules with fewer than 500 photons. Finally, the lateral drift correction was performed, and the super‐resolution image was reconstructed with a pixel size of 10 nm. To quantify the accuracy of the drift correction method, five experiments were conducted, each comprising 20 independent measurements to ensure stable evaluation results.

### Immunofluorescence Staining for Cultured Cells and Tissue Section

For immunofluorescence staining of microtubules, COS‐7 cells were pre‐extracted with cytoskeleton buffer containing 80 mm PIPES, 1 mm MgCl_2_, 5 mm EGTA supplemented with 0.5% Triton X‐100 for 30 to 60 s. The cells were fixed with absolute methanol at −20oC for 10 min, then washed with 1× PBS. The fixed cells were blocked with blocking buffer containing 3% (w/v) BSA supplemented with 0.1% (v/v) Triton X‐100 in 1× PBS for 1 h at room temperature before being incubated with rabbit anti‐α‐tubulin antibody (Abcam, ab52866) at a desired dilution (1:300) overnight at 4 °C. The cells were washed with washing buffer containing 0.2% (w/v) BSA, 0.05% (v/v) Triton X‐100 in 1xPBS for three times before being incubated with custom secondary antibody goat anti‐rabbit IgG (H+L) (Jackson ImmunoResearch, 111‐005‐144) conjugated with Alexa 647 NHS Ester (ThermoFisher, Scientific, A20106) diluted in blocking buffer for 2 h at room temperature, protected from light. For the staining of lamin B1 proteins, the cells were fixed with 4% PFA for 15 min at room temperature, being washed, and then permeabilized with 0.2% Triton X‐100 for 10 min. The fixed cells were being blocked with blocking buffer and then incubated with an anti‐lamin B1 antibody (Abcam, ab16048) at a desired dilution (1:500) overnight at 4 °C. The cells were washed and incubated with Alexa 647‐conjugated secondary antibody as described above.

For the tissue section, a 3 µm‐thick tissue section from formalin‐fixed paraffin‐embedded tissue blocks was mounted on the gelatin‐coated coverslips, deparaffinized in xylene, and rehydrated in graded ethanol, and finally stored in 1× PBS. The antigen retrieval was performed as previously described.^[^ ^28^, ^32^ ^]^ The samples were incubated with primary antibodies (H3K9me3, #ab5408, Abcam) diluted to optimized concentration at 4 °C overnight. Alexa 647‐conjugated goat anti‐rabbit secondary antibody was applied to the sample at room temperature for 2 h in the dark. The sample was washed again three times with washing buffer and once with PBS for 5 min per wash and stored in PBS before imaging. The imaging buffer was added to the sample dish right before imaging. During the image acquisition, a transparent plastic cover on top of an imaging chamber was used to minimize the exposure of the imaging buffer to air and reduce odors.

### STORM Imaging Buffer

STORM imaging buffer for cultured cells contains 10% (w/v) glucose (Sigma‐Aldrich), 0.56 mg mL^−1^ glucose oxidase (Sigma–Aldrich), 0.17 mg mL^−1^ catalase (Sigma–Aldrich), 0.14 m 2‐mercaptoethanol (βME, Sigma–Aldrich). For the FFPE tissue section, to reduce the high background caused by the strong scattering of tissue, an optical clearing process was conducted before imaging by immersing the sample in 60% (v/v) 2,2′‐thiodiethanol (TDE) for 20–30 min to make the sample transparent. For the STORM imaging buffer of FFPE tissue section, 60% (v/v) TDE solution was used instead of water to match the refractive index of tissue and contains 10% (w/v) glucose (Sigma‐Aldrich), 0.56 mg mL^−1^ glucose oxidase (Sigma–Aldrich), 0.17 mg mL^−1^ catalase (Sigma–Aldrich), 0.14 m 2‐mercaptoethanol (βME, Sigma–Aldrich), and 0.2 mm Cyclooctatetraene (COT, Sigma–Aldrich). The imaging buffer was added to the sample dish right before imaging.^[^ ^28^ ^]^

## Conflict of Interest

The authors declare no competing interest.

## Author Contributions

H.M. conceived and developed the drift correction method, built the SMLM system, and performed the experiments. P.N. prepared biological samples and performed the experiments. Y.L. developed the software for automated control of the SMLM system. H.M. and Y.L. wrote the manuscript. Y.L. acquired funding support and supervised the study. All authors discussed the results and commented on the manuscript.

## Supporting information



Supporting Information

## Data Availability

The data that support the findings of this study are available from the corresponding author upon reasonable request.

## References

[1] E. Betzig , G. H. Patterson , R. Sougrat , O. W. Lindwasser , S. Olenych , J. S. Bonifacino , M. W. Davidson , J. Lippincott‐Schwartz , H. F. Hess , Science 2006, 313, 1642. 16902090 10.1126/science.1127344

[2] S. T. Hess , T. P. K. Girirajan , M. D. Mason , Biophys. J. 2006, 91, 4258. 16980368 10.1529/biophysj.106.091116 PMC1635685

[3] M. J. Rust , M. Bates , X. Zhuang , Nat. Methods 2006, 3, 793. 16896339 10.1038/nmeth929 PMC2700296

[4] M. Heilemann , S. Van De Linde , M. Schüttpelz , R. Kasper , B. Seefeldt , A. Mukherjee , P. Tinnefeld , M. Sauer , Angew. Chem., Int. Ed. 2008, 47, 6172. 10.1002/anie.200802376 18646237

[5] A. Sharonov , R. M. Hochstrasser , Proc. Natl. Acad. Sci. 2006, 103, 18911. 17142314 10.1073/pnas.0609643104 PMC1748151

[6] J. Schnitzbauer , M. T. Strauss , T. Schlichthaerle , F. Schueder , R. Jungmann , Nat. Protoc. 2017, 12, 1198. 28518172 10.1038/nprot.2017.024

[7] H. Deschout , F. C. Zanacchi , M. Mlodzianoski , A. Diaspro , J. Bewersdorf , S. T. Hess , K. Braeckmans , Nat. Methods 2014, 11, 253. 24577276 10.1038/nmeth.2843

[8] Y. Wang , J. Schnitzbauer , Z. Hu , X. Li , Y. Cheng , Z.‐L. Huang , B. Huang , Opt. Express 2014, 22, 15982. 24977854 10.1364/OE.22.015982 PMC4162368

[9] H. Ma , M. Chen , P. Nguyen , Y. Liu , Sci. Adv. 2024, 10, 7765. 10.1126/sciadv.adm7765 PMC11114195 38781327

[10] S. P. Callahan , A. Dlasková , J. Šantorová , J. M. Schreiner , M. J. Mlodzianoski , K. Smolková , P. Ježek , J. Bewersdorf , Opt. Express 2011, 19, 15009. 21934862 10.1364/OE.19.015009

[11] J. Cnossen , J. Cnossen , T. J. Cui , C. Joo , C. Smith , C. Smith , Opt. Express 2021, 29, 27961. 34614938 10.1364/OE.426620

[12] S. Coelho , J. Baek , J. Walsh , J. J. Gooding , K. Gaus , Nat. Protoc. 2020, 16, 497. 33268882 10.1038/s41596-020-00426-9

[13] H. Ma , J. Xu , J. Jin , Y. Huang , Y. Liu , Biophys. J. 2017, 112, 2196. 28538156 10.1016/j.bpj.2017.04.025 PMC5444076

[14] P. Bon , N. Bourg , S. Lécart , S. Monneret , E. Fort , J. Wenger , S. Lévêque‐Fort , Nat. Commun. 2015, 6, 7764. 26212705 10.1038/ncomms8764 PMC4525210

[15] M. Dai , R. Jungmann , P. Yin , Nat. Nanotechnol. 2016, 11, 798. 27376244 10.1038/nnano.2016.95 PMC5014615

[16] “The Nikon Perfect Focus System (PFS) | Nikon's MicroscopyU”, https://www.microscopyu.com/tutorials/the‐nikon‐perfect‐focus‐system‐pfs.

[17] H. Ma , Y. Liu , Opt. Lett. 2021, 46, 5798. 34851893 10.1364/OL.437409 PMC8640370

[18] Y. Li , Y. He , K. Fang , L. Zhou , Z. Wang , W. Shi , W. Shi , Y. Li , Y. Li , Opt. Lett. 2024, 49, 2785. 38748161 10.1364/OL.519290

[19] K. Bellve , C. Standley , L. Lifshitz , K. Fogarty , Biophys. J. 2014, 106, 606a.

[20] A. Rahmani , A. Rahmani , T. Cox , A. T. A. Achary , A. Ponjavic , A. Ponjavic , A. Ponjavic , Opt. Express 2024, 32, 13331. 38859306 10.1364/OE.520845

[21] R. Mcgorty , D. Kamiyama , B. Huang , Opt Nanoscopy 2013, 2, 3. 10.1186/2192-2853-2-3 PMC3874277 24380058

[22] M. Shang , Z. Zhou , W. Kuang , Y. Wang , B. O. Xin , Z.‐L. Huang , Opt. Express 2021, 29, 34641. 34809249 10.1364/OE.438160

[23] M. J. Wester , D. J. Schodt , H. Mazloom‐Farsibaf , M. Fazel , S. Pallikkuth , K. A. Lidke , Sci. Rep. 2021, 11, 23672. 34880301 10.1038/s41598-021-02850-7 PMC8655078

[24] H. Ma , M. Chen , J. Xu , Y. Yang , Y. Zhao , Y. Liu , Sci. Adv. 2024, 10. 10.1126/sciadv.adq5009 PMC11482309 39413179

[25] Z. Bian , C. Guo , S. Jiang , J. Zhu , R. Wang , P. Song , Z. Zhang , K. Hoshino , G. Zheng , J. Biophotonics 2020, 13, 202000227. 10.1002/jbio.202000227 32844560

[26] J. Liao , Z. Wang , Z. Zhang , Z. Bian , K. Guo , A. Nambiar , Y. Jiang , S. Jiang , J. Zhong , M. Choma , G. Zheng , J. Biophotonics 2018, 11, 201700075. 10.1002/jbio.201700075 PMC5766431 28700137

[27] S. Koho , G. Tortarolo , M. Castello , T. Deguchi , A. Diaspro , G. Vicidomini , Nat. Commun. 2019, 10, 3103. 31308370 10.1038/s41467-019-11024-z PMC6629685

[28] J. Xu , H. Ma , Y. Liu , Curr. Protoc. Cytom. 2020, 94, e78. 32762150 10.1002/cpcy.78 PMC8622137

[29] H. Foroosh , J. B. Zerubia , M. Berthod , IEEE Trans. Image Process. 2002, 11, 188. 18244623 10.1109/83.988953

[30] H. Ma , Y. Liu , Y. Liu , Opt. Lett. 2021, 46, 3825. 34388751 10.1364/OL.433740 PMC8622170

[31] H. Ma , W. Jiang , J. Xu , Y. Liu , Sci. Rep. 2021, 11, 20417. 34650088 10.1038/s41598-021-00066-3 PMC8517018

[32] J. Xu , X. Sun , K. Kim , R. M. Brand , D. Hartman , H. Ma , R. E. Brand , M. Bai , Y. Liu , Sci. Adv. 2022, 8, 8293. 10.1126/sciadv.abm8293 PMC8896800 35245126

