# Supplementary material for: A Versatile Drift‐Free Super‐Resolution Imaging Method via Oblique Bright‐Field Correlation
Source: Adv Sci (Weinh). 2024 Dec 24;12(7):2412127. doi: 10.1002/advs.202412127 (PMC11831467; doi:10.1002/advs.202412127)
Supplement: Supplementary file 1 — Supporting Information [file ADVS-12-2412127-s001.pdf]

ADVANCED SCIENCE
Open Access

Supporting Information

for *Adv. Sci.*, DOI 10.1002/advs.202412127

A Versatile Drift-Free Super-Resolution Imaging Method via Oblique Bright-Field Correlation

Hongqiang Ma, Phuong Nguyen and Yang Liu**

A Versatile Drift-Free Super-resolution Imaging Method via Oblique Bright-Field Correlation

Hongqiang Ma[1*], Phuong Nguyen[1], Yang Liu[2*]

[1]Department of Bioengineering, The Grainger College of Engineering, Beckman Institute for Advanced Science and Technology, University of Illinois Urbana-Champaign, Urbana, IL 61801, USA

[2]Department of Bioengineering, Department of Electrical and Computer Engineering, The Grainger College of Engineering, Beckman Institute for Advanced Science and Technology, Cancer Center at Illinois, University of Illinois Urbana-Champaign, Urbana, IL 61801, USA

Corresponding author Email: *mhq@illinois.edu (H.M.); liuy46@illinois.edu (Y.L.)*

Supplementary figures

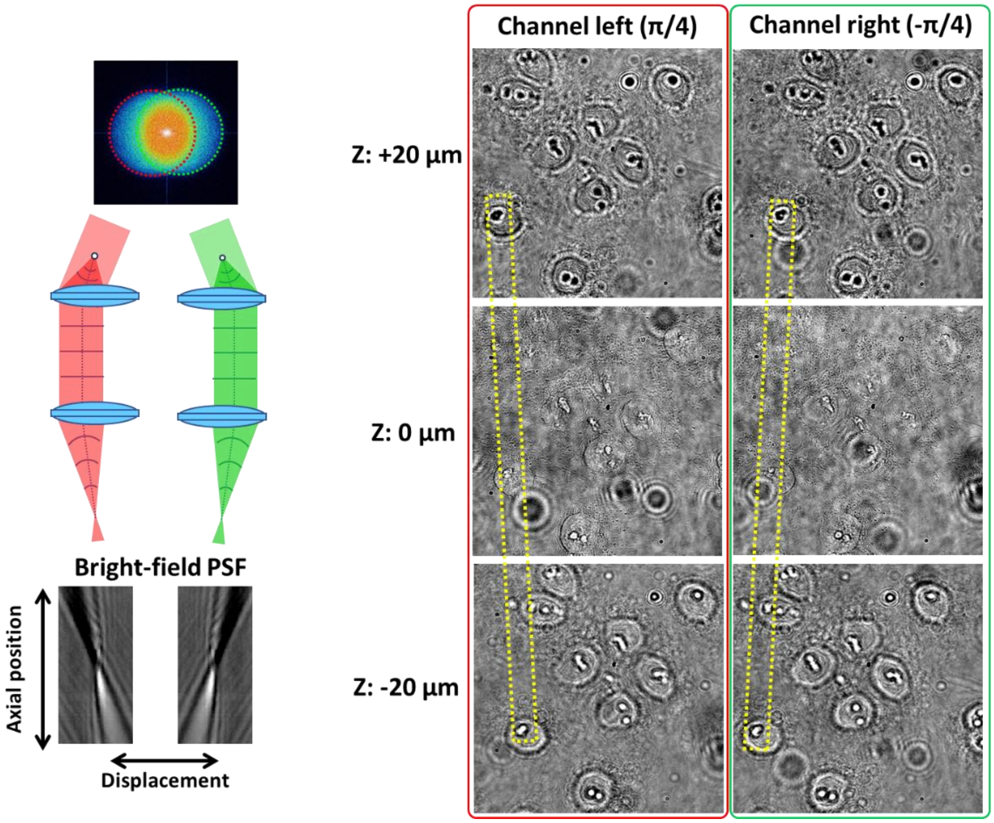

Figure S1. Illustration of the working mechanism of our method using the displacement analysis of bright-field images from the two complementary oblique illumination angles.

For bright-field imaging, a sample illuminated by a plane wave produces a total field that is the interferometric superposition of the incident plane wave and the scattered wave from the sample. According to the Fourier diffraction theorem, the scattered field encodes the spatial frequency information of the sample.

An optical wave propagating along the axial (z) axis from an in-focus plane to an out-of-focus plane at the axial position of z can be written as: $U(x, y, z) = U_0(x, y, 0)\exp(i2\pi\kappa_z z)$, where $\kappa_z = \pm\sqrt{\kappa^2 - (\kappa_x{}^2 + \kappa_y{}^2)}$. The spatial frequency component κ_z is determined by both the illumination angle and the system's pupil function. The oblique illumination angle shifts the spatial frequency spectrum in the Fourier domain, thus modifying the pupil function and introducing angle-dependent spatial frequency components, and tilting the point spread function.

In our experiment, we used two specific illumination angles, ±π/4 that shift the Fourier spectrum in the opposite directions, leading to corresponding tilts in the point spread function (PSF). The tilt of the PSF is sensitive to the axial position of sample features. As evident from the above equation, the light wave traveling out-of-focus introduces an additional phase term due to the defocus distance of z, which affects the spatial frequency distribution. By measuring the relative displacement of the PSF between images captured under the two illumination angles, we can accurately determine the axial position of the sample. This approach exploits the relationship between the illumination angle and the resulting PSF shift to extract the axial position information while maintaining the high lateral resolution derived from the shifted pupil function.

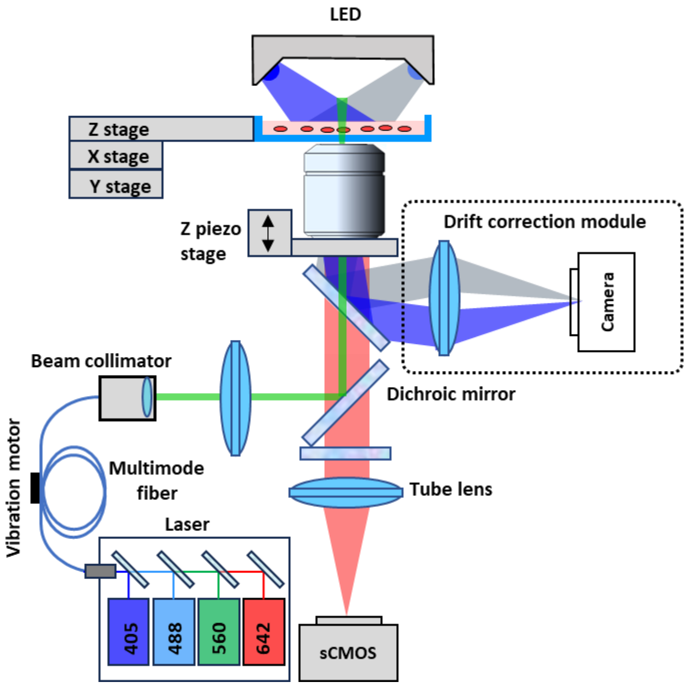

Figure S2. An alternative strategy to implement a real-time drift correction using an independent drift correction module equipped with a dedicated camera. Note that the proposed drift correction method is not restricted to specific wavelengths. Any wavelength used for bright-field imaging, including near-infrared light, can be applied here.

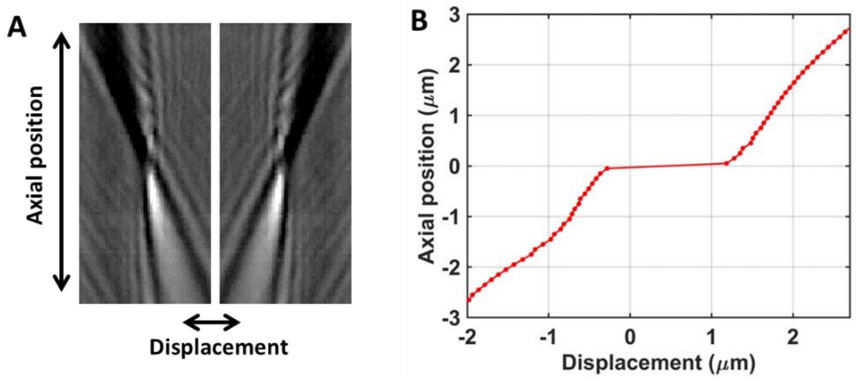

Figure S3. The point spread functions of bright-field images near the focal plane (A) to illustrate the monotonic relationship between the lateral displacement of the image features and the axial position (B).

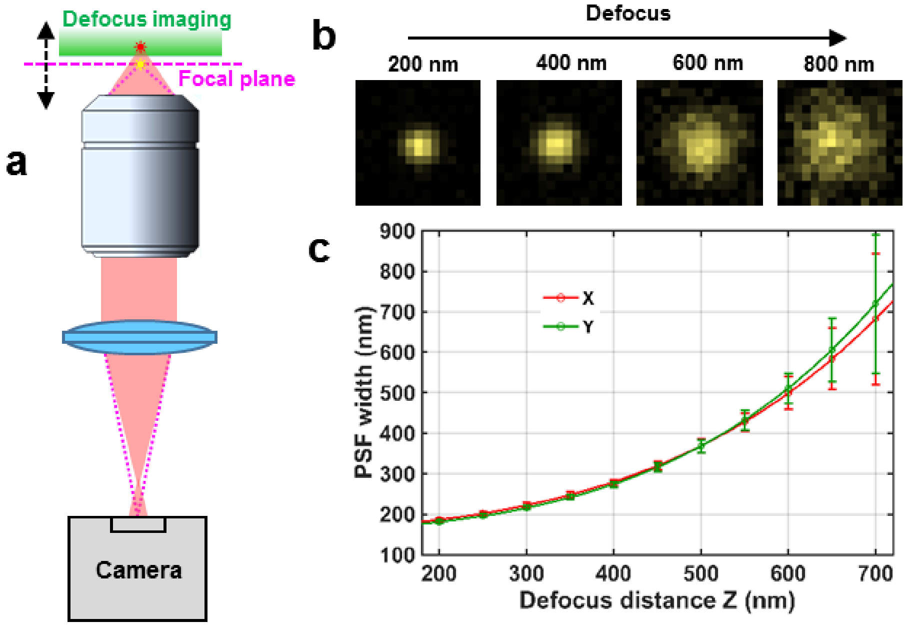

Figure S4. The principle of defocus 3D single-molecule localization microscopy (SMLM). (a) The general illustration of the defocus-based fluorescence detection in a 2D SMLM setup. (b) The point spread function (PSF) pattern of a single-molecule emitter as it deviates from the focal plane. (c) The measured width of the PSF in the x (red) and y (green) dimensions at different defocus distance Z, which is used as the calibration curve to retrieve the axial position of each fluorescent emitter. The error bar in (c) indicates standard deviation.

It is well recognized that the width of the PSF achieves the smallest value when the emitter is located at the focal plane. As the emitter defocuses from the focal plane, regardless of whether it is above or below, the width of the PSF becomes larger, as shown in Figs. 1(b-c). Therefore, the width of the PSF already encodes the axial information even on a 2D imaging system. But the exact axial position of single molecules is still unknown from a 2D imaging system, because the emitters above and below the focal plane share a similar pattern of intensity distribution. However, if the imaging object is located only at one side of the focal plane (as indicated in Fig. 1a, green region), it is possible to retrieve the exact 3D information from a standard 2D microscopy system, without the aforementioned ambiguity of their exact axial location with regard to the focal plane. The essence of the defocus microscopy is to “defocus” or move the imaging object away from the focal plane (e.g., defocus distance of 300 nm), and determine the 3D position of fluorescent emitters based on the width of PSF. This defocus-based SMLM approach can be applied to any imaging depth within the sample, provided that two conditions are met: sufficient signal-to-noise ratio and the imaging object being located at only one side of the focal plane. Since lamin B1 is positioned above the focal plane, 3D defocus-based super-resolution imaging of lamin B1 is feasible.

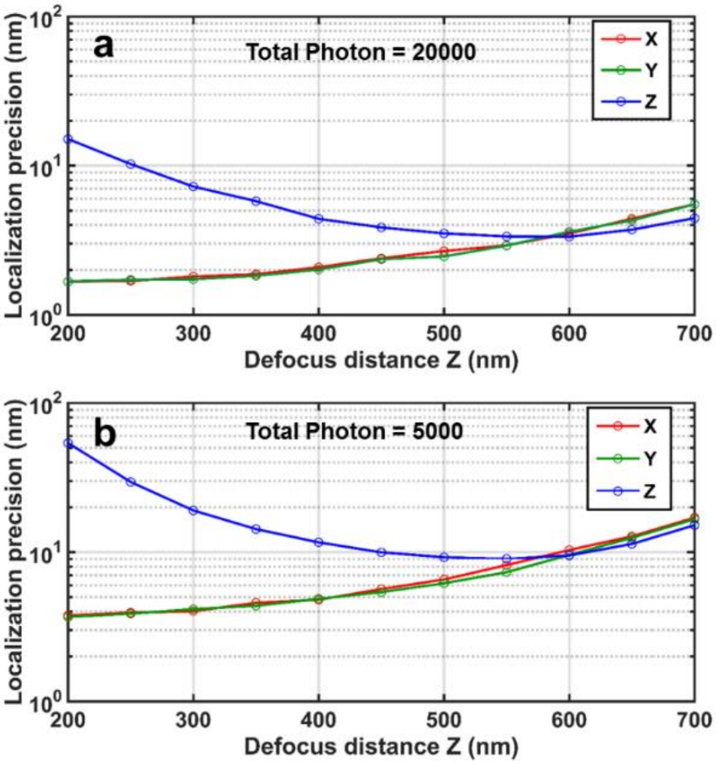

Figure S5. The performance of localization precision of our defocus-based 3D SMLM via numerical simulation. Total photon number of (a) 20,000 and (b) 5,000 photons is assumed to mimic ultra-bright fluorophores and Alexa Fluor 647, respectively. The simulated images are generated based on two scenarios where the photon count of the fluorescent emitters is 20,000 and 5,000, respectively. The former case mimics a condition with ultra-bright emitters such as fluorescent nanoparticles and the latter simulates the fluorescent properties of the commonly used Alexa Fluor 647. Our numerical simulation shows that our defocus microscopy can retrieve the exact 3D position of the single-molecule emitter from a 2D imaging system for a range of defocus distance from 200 nm to 700 nm. In the case of ultra-bright emitters with a photon number of 20,000, our defocus-based 3D SMLM achieves a localization precision of less than 7 nm in all 3D positions at the defocus distance from 300 nm to 700 nm. While in the case of commonly used photon-limited fluorophore (e.g., Alexa Fluor 647), our method achieves a localization precision of less than 10 nm in the lateral direction and 20 nm in the axial direction for a defocus distance from 300 nm to 600 nm, corresponding to their spatial resolution (defined as full width at half maximum (FWHM)) of 25 nm and 50 nm, respectively. This performance is similar to that with most conventional 3D imaging methods. Please note that, although this method theoretically retrieves the 3D positions of emitters for a long range of defocus distance, in the case of super-resolution localization imaging experiment with photon-limited fluorophores (e.g. Alexa Fluor 647), only the defocus distance from 300 nm to 600 nm are recommended. For defocus distance close to the focal plane (<300nm), the width of PSF changes little over the axial position, hence compromising the localization precision in the axial direction (larger than 20 nm).

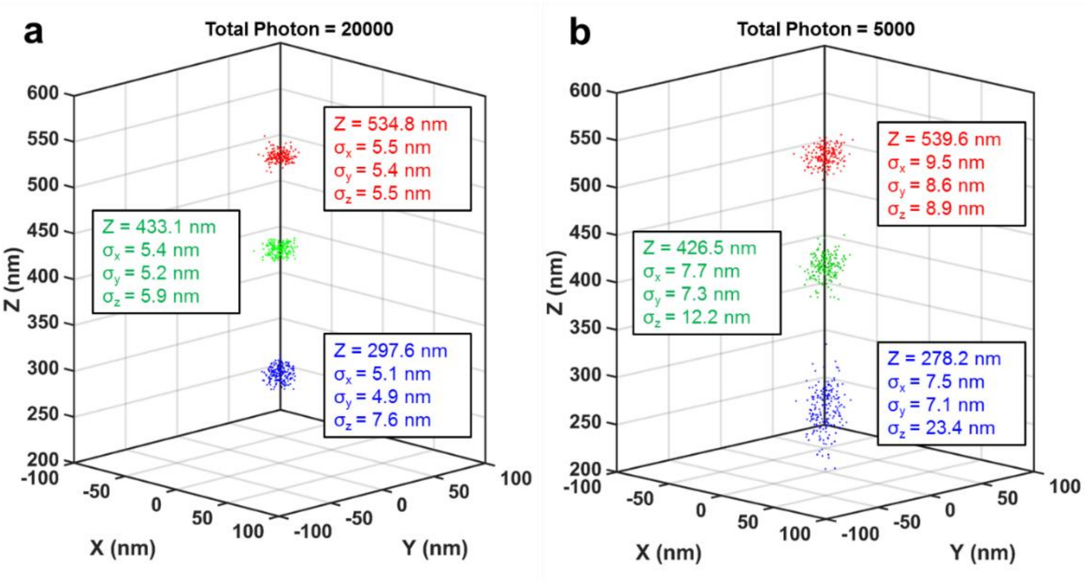

Figure S6. Performance of our defocus-based 3D SMLM for 3D localization imaging experiments using fluorescent nanoparticles. (**a-b**) The retrieved 3D positions of the fluorescent nanoparticles and the localization precision in all three dimensions. It shows a localization precision of ~4-8 nm in 3D positions in the case of a high photon number; and a localization precision of less than 10 nm in the lateral direction and ~8-23 nm in the axial direction in the case of a photon-limited emitter, in agreement with the results from numerical simulations shown in Fig. S2.

Fluorescent nanoparticles with an average photon number of (a) 20,000 and (b) 5000 photons are selected to mimic ultra-bright fluorophores and Alexa Fluor 647, respectively for subsequent analysis. We captured the image stacks of single fluorescent nanoparticles (100 nm diameter), at the defocus distance of around 300, 450 and 550 nm. At each position, we captured 200 images with a frame rate of 25 fps, and an average photon number of approximately 20,000 and 5,000 per localization, respectively.
